
JOURNAL INFORMATION
==============================
NLM Title Abbreviation: Tex Med J (Austin)
NLM Journal ID: 21130590R

Texas Medical Journal

ISSN: 0892-8495
Publisher: Biomedical Journal Digitization Project

ARTICLE INFORMATION
==============================
PMCID: PMC9590904
Article version: 1
Subjects: Books and Magazines

Atlas and Essentials of Pathological Anatomy

Print publication date: 1899 Jan
Volume: 14
Issue: 7
First page: 410
Last page: 410
License: This work is free of known copyright restrictions. For more information, please see PMC Back Issue Digitization.
License URL: https://creativecommons.org/publicdomain/mark/1.0/

==============================

Atlas and Essentials of Pathological Anatomy.—By Dr. O. Ballinger, Obermedicinalrat and Professor. Vol. I. Circulatory, respiratory and digestive apparatus, including the liver, bile ducts and pancreas, with 69 colored figures upon 60 plates and 18 illustrations in the text. Published by William Wood & Co., New York. 1898. Price, cloth, $3.00 net.

The first volume of this work, a continuation of the series of Wood's Hand Atlases, a storehouse of beautiful art and useful information.

I fear that it is not possible to convey in a short descriptive article the beauty and value of these hand atlases. Suffice to say that each of these sixty colored plates is a separate work of art of the highest grade, and of sufficient size to illustrate the subject in all its details. The descriptive text is on the page opposite the plate, and taken together they fully elucidate the subject.

In addition to the plates and descriptive text, there is appended a concise treatise on pathological anatomy of 246 pages. Too much cannot be said in commendation of the publishers' enterprise in presenting the work to the profession at the remarkably low price. It is only to be regretted that the books are not bound in durable leather.

I. J. J.

---

### Publisher's Notes.

Dr. J. W. Pendergast, of the Department of Health, Cincinnati, O., in a recent letter writes, concerning Wayne's Elixir, that he has been using it in his practice for many years, and when a diuretic is indicated that there is no preparation, with which he is familiar, that meets the requirements so well. He feels that he is but doing his duty to recommend to the profession a remedy of such known value.

---

New Orleans Polyclinic.—Physicians will find the Polyclinic an excellent means for posting themselves upon modern progress in all branches of medicine and surgery. The specialties are fully taught, particularly laboratory work. The 12th Annual Session opens November 24th, 1898. For further information, address New Orleans Polyclinic, P. O. Box 797, New Orleans, La.

---

The American Navy, Cuba and Hawaii.—A portfolio, 10½x14 inches, in *ten parts*, sixteen views in each part, of the finest halftone pictures of the American Navy, Cuba and Hawaii, has just been published, and the Gulf, Colorado and Santa Fe Railway has made arrangements for a special edition for the benefit of its
